# Letter from M. Heyfelder on Disarticulation of the Lower Jaw

**Published:** 1859-01

**Authors:** 


### ARTICLE V.

*Letter from* M. HEYFELDER *on Disarticulation of the Lower Jaw.* Translated for the American Journal of Dental Science.

M. HEYFELDER, Senior, corresponding member of the Surgical Society of St. Petersburg, has written the following letter upon the question of Disarticulation of the Jaw, which has recently been discussed by the Medical Society. of Paris.

"I have followed the discussion on this operation with much interest, and as I have practiced disarticulation of the jaw in Germany, Finland and here almost as often as M. Maissonneuve, I ask permission to communicate to the society the results of my observations. In the first place there are two questions to settle, viz. 1st. Is it preferable to perform the operation by dissecting out with the bistoury (and scissors) all the muscular insertions and soft parts which are attached to the inferior maxillary bone, or to destroy all the attachments by a manœuvre composed of tractions and torsions which has been named extractial? 2d. Does one always succeed without difficulty in detaching the temporal muscle from the coronoid apophysis, or is it necessary sometimes to cut this apophysis at its base with the assistance of Liston's cutting pincers, separated and afterwards secured by hook pincers and using Cooper's scissors?

I believe that all surgeons will fall into my belief when I say, that the detachment of the tendon of the temporal muscle of the coronoid apophysis is the only portion of the operation which presents any difficulty.

M. Chassaignac has proved before the society that the length of the apophysis is not always the same, and that they differ sufficiently often in the ordinary length. I have observed the same thing, but principally here at St. Petersburg indifferently upon the living and dead subjects upon whom I performed the operation. In most cases I succeeded in detaching the tendon with the bistoury or with

Cooper's scissors, holding the jaw down with the fingers of the left hand ; but two or three times this was not sufficient and I was obliged to cut the base of the apophysis with the cutting pincers, a not very easy thing when the bone is very hard. However, I confess I know of no other instrument for this purpose preferable to Liston's pincers. Once M. Ried, professor of clinical surgery at Jena, was present when I used the cutting pincers, and he acknowledged that at first he had considered the proceeding as superfluous but that this case proved the contrary. So that I am of the opinion that it is necessary always to separate the cut apophysis, which is better done with a pair of hooked pincers and using Cooper's scissors. The detachment of the condyle from the soft parts does not present the same difficulties. Here one is able to cut with the bistoury and the scissors all the muscular insertions and open the capsular ligament of the condyloid apophysis, after which a slight traction generally suffices to make the separation complete, a process which should not be called a process of rooting out or extraction. It has happened to me more than once to find that the bones became fractured while I was removing the soft parts, still I did not find that this accident much increased the difficulty of the operation. In two cases in which I made the complete ablation of the jaw the bone broke immediately at the two condyles, nevertheless I succeeded in removing them with little trouble. The process of extraction when thus practiced does not produce gangrene of the wound, an event which I have met with only once, but at a time when all the wounds sphacelated more or less easily. Consecutive hemorrhages have been spoken of also. These hemorrhages occur where a small artery is wounded very near its origin from the carotid, when the ligature which is put on comes away, and when the patient first masticates bread or meat. In this way I have seen a patient die three days after the operation, the attending physician neglecting to ligature the carotid which I would have done if I had been present."

[*L'Art Dentaire.*

